# Pathological characteristics and predictive factors of prostate biopsy in patients with serum PSA levels between 0 and 4.0 ng/ml

**DOI:** 10.3389/fonc.2022.957892

**Published:** 2022-07-28

**Authors:** Rui Su, Jin-feng Pan, Da-wei Ren, Jun-hui Jiang, Qi Ma

**Affiliations:** ^1^ Comprehensive Urogenital Cancer Center, Ningbo First Hospital, The Affiliated Hospital of Ningbo University, Ningbo, China; ^2^ Department of Urology, Ningbo First Hospital, The Affiliated Hospital of Ningbo University, Ningbo, China; ^3^ Ningbo Clinical Research Center for Urological Disease, Ningbo, China; ^4^ Medical School, Ningbo University, Ningbo, China; ^5^ Department of Radiology, Ningbo First Hospital, The Affiliated Hospital of Ningbo University, Ningbo, China; ^6^ Translational Research Laboratory for Urology, The Key Laboratory of Ningbo City, Ningbo First Hospital, The Affiliated Hospital of Ningbo University, Ningbo, China

**Keywords:** prostate tumor, prostate biopsy, multiparametric magnetic resonance imaging, PI-RADS score, prostate-specific antigen

## Abstract

**Background:**

This study aimed to analyze the pathological characteristics and predictive factors of prostate biopsy in men with PSA levels below 4.0 ng/ml.

**Patients and methods:**

We retrospectively analyzed 158 patients who underwent prostate biopsy with PSA levels below 4.0 ng/ml. Pathological results were statistically analyzed. The logistic regression analysis was used to determine the predictive factors for malignant outcomes. Subgroup analysis was performed on patients who received surgery and the postoperative pathological upgrading was counted.

**Results:**

A total of 143 patients were enrolled. The tumor detection rate was 20.3%. Among these patients, most of them (79.3%) had prostate adenocarcinoma, but rare malignant tumors also accounted for 20.7%. Logistic regression analysis indicated that the only independent predictive factor for a positive prostate biopsy was the PI-RADS score. For prostate adenocarcinoma cases, 95.7% of them were organ localized and 47.8% of cases were clinically significant. Subgroup analysis was performed on 14 patients who received surgical treatment. 28.6% of patients were upgraded to clinically significant prostate cancer, while 64.3% of patients had an upgrade in tumor stage.

**Conclusion:**

Our study indicated that 20.3% of men with PSA levels between 0 and 4.0 ng/ml were diagnosed with prostate malignancies. Among these patients, most of them (79.3%) were diagnosed with prostate adenocarcinoma, and several uncommon types of malignancies were also detected in 20.7% of patients. The only risk factor for a positive biopsy in patients with a low PSA concentration was the PI-RADS score. It should be emphasized that the invasiveness of PCa patients diagnosed by biopsy may be underestimated as more than half of patients will upgrade their Gleason score or clinical stages after surgery. Thus, clinicians should pay more attention to patients with PSA levels between 0 and 4.0 ng/ml.

## Introduction

Prostate cancer (PCa) is the second leading cause of cancer death in American men (1). There were expected to be about 174,650 new PCa diagnoses and about 31,620 deaths in the United States in 2019 (2). Prostate biopsy is the gold standard for the diagnosis of PCa (3). The need for prostate biopsy is based on a PSA level and/or suspicious digital rectal examination (DRE) and/or abnormal imaging (4). A serum PSA level exceeding 4.0 ng/ml is considered to have a diagnostic value for PCa (5). However, a biopsy could still be performed in patients with a low PSA level between 0 and 4.0 ng/ml due to abnormal magnetic resonance imaging (MRI), ultrasound, or DRE.

It was reported that PCa was not rare in patients with a PSA level of 4.0 ng/ml or less. Some investigators have suggested that 2.5 ng/ml may be a more appropriate cut point than 4.0 ng/ml (6, 7). On the other hand, several studies have focused on finding the risk factors to predict positive biopsy results among patients with low PSA levels. Eastham et al. (8) reported serum PSA was the only independent predictor of positive prostate biopsy in the multivariate analysis of prebiopsy risk factors (age, race, PSA). Djavan et al. (9) prospectively evaluated various PSA-based diagnostic parameters in men with PSA levels of 2.5 to 4.0 ng/ml. It was found that compared with standard total PSA assays, the free/total PSA ratio (f/tPSA) and PSA density of the transition zone (PSA-TZ) significantly enhance the sensitivity and specificity of PCa detection in a patient population with a total PSA of 2.5 to 4.0 ng/ml.

However, the incidence of PCa shows regional and racial differences. The findings of American and European populations may not be suitable for Chinese men. The histopathological findings, tumor characteristics, and independent risk factors of Chinese patients with PSA levels below 4.0 ng/ml have not been well described. Moreover, multiparametric MRI (mpMRI) has been recommended by the European Association of Urology (EAU) guidelines since 2019, and we could forecast the detection rate of clinically significant prostate cancer (csPCa) using prostate imaging reporting and data system (PI-RADS) scores (10, 11). As a useful tool, the role of the PI-RADS score in predicting positive biopsy with low PSA has not been fully evaluated. Therefore, we retrospectively analyzed the pathological findings and clinical parameters (age, PI-RADS score, TRUS, and PSA-based parameters) of prostate biopsy cases with PSA levels between 0 and 4.0 ng/ml. As this study belonged to a series of studies performed in our comprehensive urogenital cancer center, we named this study YH-prostate-002. The aim of this study was to analyze the pathological characteristics and predictive factors of prostate biopsy in men with low PSA concentration.

## Materials and methods

### Study design

**Figure 1:**
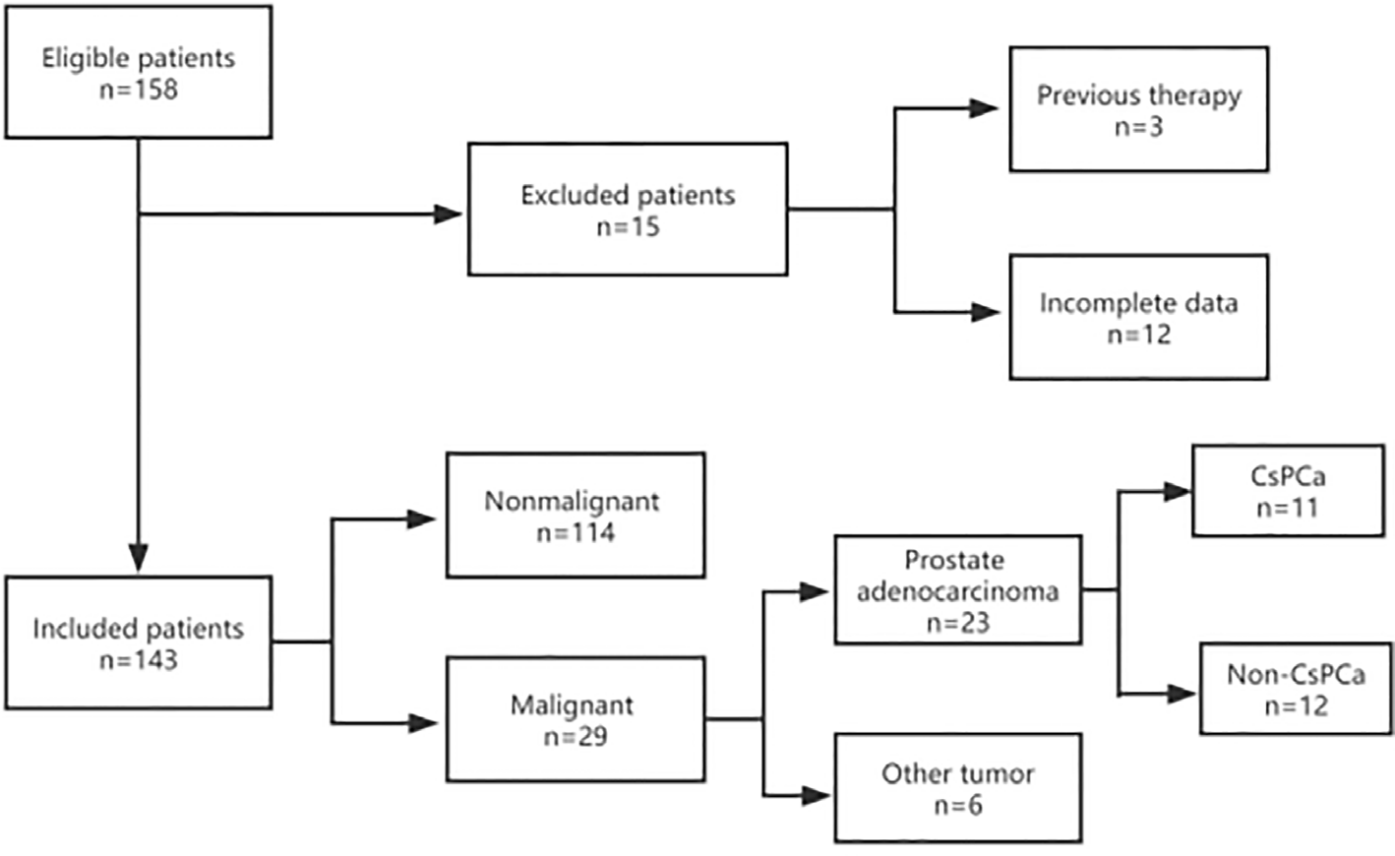
Flow diagram of the inclusion and exclusion criteria in this study. A total of 143 patients were enrolled in this study. All patients were recorded with complete information, including mpMRI, TRUS, DRE, age, tPSA, and f/tPSA. Prostate volume (PV) and PSAD were also collected and measured before prostate biopsy. After a biopsy, the pathological results were collected and recorded. If the patients accepted radical proctectomy, postoperative pathology was also collected and recorded.

Between January 2014 and December 2021, a total of 158 men with a serum PSA level of less than 4.0 ng/ml underwent transrectal ultrasound (TRUS)-guided prostate biopsy in Ningbo First Hospital, China. They were initially suspected of prostate cancer because of abnormal DRE and/or TRUS results during routine examinations in the urological inpatient or outpatient department. Due to the high diagnostic value of mpMRI, most of them received further mpMRI scanning to make the biopsy more accurate. Inclusion criteria were complete mpMRI, TRUS, and DRE result data before a biopsy. Clinical parameters such as age, tPSA, f/tPSA, PSA density (PSAD), and prostate volume (PV) were also measured and recorded. Exclusion criteria include a history of drug use (e.g., 5 α-reductase inhibitors) or prostate surgery prior to biopsy and incomplete data (**Figure 1**). All patients provided written informed consent before biopsy.

### mpMRI protocol and PI-RADS score

All mpMRI examinations were performed before biopsy with the Siemens Magnetom Sonata 1.5T or Vida 3.0T superconducting MR scanner, using body array coils (no endorectal coil). T1WI, T2WI, DWI (ADC), and enhancement sequence were involved in the routine scan protocol. The MRI images of enrolled cases were retrieved from the local database of the hospital. These data were reviewed again by one urologist (RS) and one radiologist (DWR), who were experienced and blinded to pathological results. The PI-RADS score was performed according to the PI-RADS v.2 scoring protocol (12). The different results of the PI-RADS score were evaluated and discussed again until an agreement was reached.

### TRUS and definition

TRUS examinations were conducted on each patient before a biopsy. Hypoechoic lesions and/or microcalcifications in the peripheral zone of the prostate were defined as positive. The PV was calculated by TRUS using the formula PV = Transverse diameter × anteroposterior diameter × cephalocaudal diameter × 0.52. The PSAD indicated the ratio of PSA to PV.

### Biopsy procedure and histopathology

TRUS-guided biopsy procedures were performed by an experienced physician (RS) using a 7.5-MHz endocavity ultrasonic probe with the MyLab40 (Esaote Biomedical, Genoa, Italy) or HI-VISION Preirus (Hitachi Medical, Tokyo, Japan) ultrasound system. The biopsy was initially performed using the transrectal approach and was switched to the transperineal approach in September 2018. Four to 14 core-targeted or systematic biopsy samples were obtained using an 18-gauge biopsy needle. All samples were sent to Ningbo Pathology Center for pathological diagnosis, using the 2014 International Society of Urological Pathology (ISUP)-modified Gleason grading system. Furthermore, according to the recommendation of the EAU Guidelines on Prostate Cancer (4), Gleason score of > 6 (ISUP ≥ 2) was defined as csPCa.

### Statistics

The basic characteristics, clinical information, and pathological results of patients were summarized by descriptive statistics. The detection rate of prostate tumors according to the PSA range (0.0–2.5 vs. 2.6–4.0 ng/ml) was compared using the Chi-square test or Fisher’s exact test. All mpMRI results and the pathological results according to different PI-RADS scores for all patients were also statistically stratified. Furthermore, a univariate logistic regression analysis was used to screen whether a positive biopsy result was associated with PI-RADS score, TRUS, DRE, age, PSA, f/tPSA, PV, and PSAD. A multivariate logistic regression analysis was then performed to determine the independent risk factors for positive biopsy results. In addition, we analyzed the patients with PCa who underwent radical prostatectomy and recorded the postoperative pathological results. Statistical analyses were performed with IBM SPSS (version 20.0). A *p*-value < 0.05 was considered statistically significant.

## Results

### Patient characteristics and pathological results

Finally, a total of 143 patients were enrolled in this study. The mean age was 65 years, with a median age of 64 years (range, 31–85). The mean PSA was 2.16 ng/ml, with a median of 2.21 ng/ml (range, 0.06–3.95). The mean volume of the prostate was 35.7 cm^3^, with a median of 31.9 cm^3^, ranging from 12 to 88 cm^3^. The mean number of cores taken in each biopsy was 9.5, with a median of 10, ranging from 4 to 14. The median PI-RADS score was 3 (range, 2–5).

**Figure 2:**
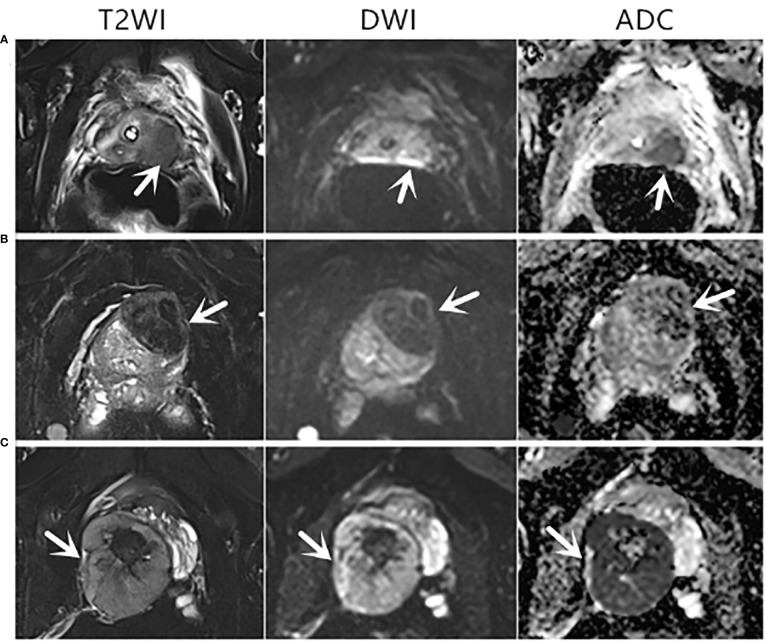
Axial mpMRI images of rare prostate tumor lesions. The mpMRI imaging of three patients who were diagnosed with rare prostate tumors. The PI-RADS score of three patients was 5. **(A)** An 82-year-old patient with a PSA level of 0.37 ng/ml and urothelial carcinoma revealed by biopsy pathology. **(B)** A 68-year-old patient with a PSA level of 1.24 ng/ml and a solitary fibrous tumor revealed by biopsy pathology. **(C)** A 31-year-old patient with a PSA level of 0.73 ng/ml and sarcoma revealed by biopsy pathology. T2WI, T2-weighted image; DWI, diffusion-weighted imaging; ADC, apparent diffusion coefficient.

Among the 143 patients, 29 cases had malignant results, and the tumor detection rate was 20.3%. In addition to the 23 cases of prostate adenocarcinoma, there was one case each of sarcoma, mucinous adenocarcinoma, neuroendocrine carcinoma, lymphoma, urothelial carcinoma, and solitary fibrous tumor (**Figure 2**). The pathological results of all patients after prostate biopsy are listed in **Table 1**. The detection rate of rare prostate tumors in the 0.0–2.5-ng/ml group was significantly higher than that in the 2.6–4.0-ng/ml group. There was no significant difference in the detection rate of prostate adenocarcinoma and all prostate malignancies between the two groups (**Table 2**). In addition, we also statistically stratified mpMRI results and the malignant pathological results according to different PI-RADS scores (**Table 3**). If the PI-RADS score was 4, the likelihood of being diagnosed as a prostate tumor was 41.7%, and if the PI-RADS score was 5, the likelihood of being diagnosed as a prostate tumor was 100%.

**Table 1:** Diagnosis of prostate biopsy in 143 patients with PSA levels below 4.0 ng/ml.

Group	Diagnosis	*n*
Malignant	Prostate adenocarcinoma	23
	Sarcoma	1
	Mucinous adenocarcinoma	1
	Neuroendocrine carcinoma	1
	Lymphoma	1
	Urothelial carcinoma	1
	Solitary fibrous tumor	1
Nonmalignant	Benign	104
	ASAP	3
	HGPIN	4
	ASAP+HGPIN	1
	Granulomatous prostatitis	2
Total		143

ASAP, atypical small acinar proliferation; HGPIN, high-grade prostatic intraepithelial neoplasia.

**Table 2:** Comparison of prostate tumor detection rate between patients with PSA levels of 0.0–2.5 and 2.6–4.0 ng/ml.

PSA (ng/ml)	Cases (*n*)	Prostate tumor detection rate (%)		
		Adenocarcinoma	Other tumors	All malignancies
0.0–2.5	81	9 (11.1)	6 (18.5)	15 (18.5)
2.6–4.0	62	14 (22.6)	0 (0.0)	14 (22.6)
*p*-value	–	0.064	0.036^*^	0.549
Total	143	23 (16.1)	6 (4.2)	29 (20.3)

^*^ p-value < 0.05 was considered statistically significant.

**Table 3:** Malignant pathological results of different PI-RADS scores.

PI-RADS score	Cases (*n*)	Malignant pathological results (*n*)				Positive rate (%)
		csPCa	Non-csPCa	Other	Total	
2	23	2	2	0	4	17.4
3	103	6	8	1	15	14.6
4	12	3	1	1	5	41.7
5	5	1	0	4	5	100
Total	143	12	11	6	29	20.3

csPCa, clinically significant prostate cancer.

### Predictive factors

In univariable analysis, PI-RADS score (*p* = 0.001), TRUS (*p <* 0.001), and age (*p* = 0.005) were significant predictors for malignant outcome, except for DRE, PSA, f/tPSA, PV, and PSAD (*p* > 0.05) (**Table 4**). However, a multivariate analysis of screened prebiopsy risk factors (PI-RADS score, TRUS, age) showed that the PI-RADS score was the only independent predictive factor for malignancy results of prostate biopsy (**Table 5**). A 1-point increase in PI-RADS score was associated with a 2.21-fold increase in the likelihood of a positive biopsy. PI-RADS score interactions with TRUS (*p* = 0.774) and age (*p* = 0.126) were not significant.

**Table 4:** Univariable logistic regression analysis to screen the predictive factors for the positive outcomes of prostate biopsy.

Indicators	B	SE	*p*-value
PI-RADS score	1.228	0.362	0.001^*^
TRUS	−1.571	0.275	<0.001^*^
DRE	0.894	0.665	0.179
Age	−4.817	1.715	0.005^*^
PSA	0.108	0.187	0.562
f/tPSA	−1.499	2.021	0.458
PV	0.008	0.012	0.510
PSAD	−0.510	5.092	0.920

^*^ p-value<0.05 was considered statistically significant.

**Table 5:** Multivariate logistic regression analysis to determine the predictive factors for the positive outcomes of prostate biopsy.

Indicators	B	SE	*p*-value	OR (95%CI)
PI-RADS score	1.166	0.377	0.002^*^	3.210 (1.534–6.717)
TRUS	0.137	0.478	0.774	1.147 (0.450–2.925)
Age	0.038	0.025	0.126	1.038 (0.989–1.090)

^*^ p-value < 0.05 was considered statistically significant.

### Postoperative pathological upgrading

According to the data of prostate adenocarcinoma cases, 95.7% of them were organ localized, while 47.8% (11/23) of the cases had Gleason score of >6 (ISUP ≥ 2) and were classified as clinically significant (**Table 6**). Subgroup analysis was performed on 14 patients who received surgical treatment; 28.6% (four of 14) of patients were upgraded to clinically significant prostate cancer, while 64.3% (nine of 14) of patients had an upgrade in tumor stage (**Table 7**).

**Table 6:** Tumor characteristics of prostate adenocarcinoma patients with PSA levels below 4.0 ng/ml.

Variables	Diagnosis	*n*
Gleason score	6	12
	7	8
	>7	3
Stage	T2aN0M0	11
	T2bN0M0	4
	T2cN0M0	7
	T3bN0M0	1
Total		23

**Table 7:** Pathological upgrading of prostate adenocarcinoma patients undergoing surgery with PSA levels below 4.0 ng/ml.

Number of patients	Gleason score		Stage		Upgrade to csPCa	Upgrade of stage
	Biopsy	Surgery	Biopsy	Surgery		
1	6	7	T2aN0M0	T2cN0M0	Y	Y
2	6	7	T2aN0M0	T2cN0M0	Y	Y
3	6	7	T2aN0M0	T2cN0M0	Y	Y
4	6	8	T2aN0M0	T2cN0M0	Y	Y
5	6	6	T2aN0M0	T2cN0M0	N	Y
6	6	6	T2aN0M0	T2cN0M0	N	Y
7	6	6	T2aN0M0	T2cN0M0	N	Y
8	7	7	T2aN0M0	T3aN0M0	N	Y
9	9	9	T2bN0M0	T2cN0M0	N	Y
10	6	6	T2aN0M0	T2aN0M0	N	N
11	6	6	T2cN0M0	T2cN0M0	N	N
12	6	6	T2cN0M0	T2cN0M0	N	N
13	7	7	T3bN0M0	T3bN0M0	N	N
14	7	7	T2cN0M0	T2cN0M0	N	N

csPCa, clinically significant prostate cancer; Y, yes; N, no.

## Discussion

The serum PSA is an important biomarker that correlates with the risk of PCa (13). In general, a PSA level of > 4 ng/ml is considered abnormal. Over the past few decades, it was reported that prostate malignancy with a PSA concentration of below 4.0 ng/ml was not rare, with the detection rate of PCa ranging from 17% to 32% (14–16). However, few studies have reported the possibility of other malignant outcomes in biopsy patients with low PSA levels.

In the present study, we found that the detection rate of prostate adenocarcinoma was 16.1% (23/143). In addition, we also diagnosed each case with sarcoma, mucinous adenocarcinoma, neuroendocrine carcinoma, lymphoma, urothelial carcinoma, and solitary fibrous tumor, accounting for 20.7% (6/29) of all positive patients. A solitary fibrous tumor is a borderline tumor with malignant potential, so we classified it into the malignant group in this study (17). Thus, the total malignant tumor detection rate was 20.3% (29/143). These malignancies are rare in the prostate and usually do not lead to abnormal elevation of PSA. In this study, they mainly appeared in the very low PSA (0.0–2.5 ng/ml) group. Therefore, we believe that for patients with PSA of less than 4 ng/ml, the purpose of biopsy should not only be to diagnose adenocarcinoma but also all possible tumors, and the study of risk factors should also include these rare malignant pathological results.

On the other hand, some published reports now support a PSA threshold of 2.5 ng/ml for recommending prostate biopsy (6, 16, 18). However, in this study, there was no significant difference in the detection rate of prostate adenocarcinoma and all prostate malignancies between the 0.0–2.5- and 2.6–4.0-ng/ml groups. Moreover, rare tumors appear to be more likely to occur in the 0.0–2.5-ng/ml group. Therefore, the optimal cutoff value of PSA is still controversial, and the predictive value of PSA is questionable in a low PSA (<4 ng/ml) population.

MpMRI is currently the best imaging method for detecting PCa (19). The PI-RADS score is widely used in clinical practice to assess the risk of PCa based on mpMRI (12). However, to the best of our knowledge, the PI-RADS score has never been included in the analysis of risk factors in the past studies among low PSA populations. Our study indicated that the PI-RADS score was the only risk factor for a positive prostate biopsy in patients with PSA of less than 4 ng/ml. A 1-point increase in PI-RADS score was associated with a 2.21-fold increase in the likelihood of a positive biopsy, and the tumor detection rate of PI-RADS scores 4 (41.7%) and 5 (100%) is very high. This result is different from previous studies. Chang et al. and Sasaki et al. reported that f/tPSA could be utilized as an effective predictor for PCa, especially in patients with PSA levels of 4 ng/ml or less (20, 21). In addition, a low f/tPSA was remarkably associated with a Gleason score of 7 or higher (20). Measurement of f/tPSA might enhance the detection of high-grade cancer that warrants aggressive treatment (21). Another study included 273 consecutive men with serum PSA of 2.5 to 4.0 ng/ml referred for early PCa detection. PSA, PSAD, PSA-TZ, f/tPSA, and PSAV were evaluated, and it was found that f/tPSA and PSA-TZ were the most powerful predictors of PCa (9). We suppose that the differences between our study and previous reports may be due to the following three reasons: (1) mpMRI was seldom included in previous studies. However, compared with PSA-based parameters, mpMRI has better prediction efficiency (22, 23); (2) previous studies mainly discussed the detection of prostate cancer, while this study discussed the predictors of all malignant tumors; and (3) tumor characteristics show regional and racial differences.

In terms of tumor invasiveness, almost all prostate adenocarcinomas in this study were organ localized (95.7%), and clinically significant prostate cancer accounted for 47.8%. This result is consistent with some previous studies. PCa patients with a PSA level of < 4 ng/ml were considered to have more favorable tumor characteristics at diagnosis (24). Biopsy in men with PSA levels between 2.6 and 4.0 ng/ml may detect clinically significant prostate cancer more frequently at an organ-confined stage, with a lower PSA progression rate (25). Furthermore, Shao et al. reported that low-risk cancers accounted for about 54% of PCas detected in patients with PSA levels of 4.0 ng/ml or less (26). However, it is worth noting that in the subgroup analysis of 14 patients undergoing radical prostatectomy, 28.6% (4/14) of patients were upgraded to clinically significant prostate cancer, while 64.3% (9/14) of patients had an upgrade of tumor stage. Therefore, we believe that the invasiveness of PCa patients diagnosed by biopsy with PSA levels between 0 and 4.0 ng/ml may be underestimated and should be paid more attention to by clinicians.

## Conclusions

To our knowledge, this report is one of the few studies focused on prostate biopsy with PSA levels between 0 and 4.0 ng/ml in China, and this study has the largest amount of patients enrolled with mpMRI detections in a single center. Our preliminary study indicated that 20.3% of men with PSA levels between 0 and 4.0 ng/ml were diagnosed with prostate malignancies. Among these patients, most of them (79.3%) were diagnosed with prostate adenocarcinoma, and several uncommon types of malignancies were detected in 20.7% of patients. Therefore, the purpose of prostate biopsy in these patients should not only be to diagnose adenocarcinoma but also all possible tumors. The only independent predictive factor for a positive biopsy in patients with a low PSA concentration was the PI-RADS score. If the PI-RADS score is ≥ 4, more than half of the patients will be diagnosed with prostate tumors after biopsy. Furthermore, it should be emphasized that after surgery, about 28.6% of patients had an upgrade in Gleason score and 64.3% (9/14) of patients had an upgrade in tumor stage. Thus, the invasiveness of PCa patients diagnosed by biopsy may be underestimated and should be paid more attention to by clinicians.

## Data Availability Statement

The original contributions presented in the study are included in the article/supplementary material. Further inquiries can be directed to the corresponding authors.

## Ethics Statement

The studies involving human participants were reviewed and approved by the ethical review committee of Ningbo First Hospital. Written informed consent for participation was not required for this study in accordance with the national legislation and the institutional requirements.

## Author Contributions

Conception/design: RS and QM. Provision of study materials or patients: J-hJ and QM. Collection and/or assembly of data: RS, J-fP, and D-wR. Prostate biopsy: RS. Data analysis and interpretation: RS and QM. Manuscript writing: RS and QM. Final approval of manuscript: All authors. All authors contributed to the article and approved the submitted version.

## Funding

This study was supported by the Zhejiang Natural Science Fund (Grant No. LY20H050002 to QM), the Medical Health Science and Technology Project of Zhejiang Provincial Health Commission (Grant No. 2021KY977 to RS), Ningbo Social Development Fund (Grant No. 202002N3192 to QM), and the Fund of Ningbo Clinical Research Center for Urological Disease (2019A21001).

## Conflict of Interest

The authors declare that the research was conducted in the absence of any commercial or financial relationships that could be construed as a potential conflict of interest.

## Publisher’s Note

All claims expressed in this article are solely those of the authors and do not necessarily represent those of their affiliated organizations, or those of the publisher, the editors and the reviewers. Any product that may be evaluated in this article, or claim that may be made by its manufacturer, is not guaranteed or endorsed by the publisher.
